# Women With Osteoarthritis Are at Increased Risk of Ischemic Stroke: A Population-Based Cohort Study

**DOI:** 10.2188/jea.JE20200042

**Published:** 2021-12-05

**Authors:** Chung-Hsin Yeh, Wei-Lun Chang, Po-Chi Chan, Chih-Hsin Mou, Ko-Shih Chang, Chung Y. Hsu, Shiow-Luan Tsay, Min-Tein Tsai, Min-Hsien Hsu, Fung-Chang Sung

**Affiliations:** 1 Department of Nursing, College of Nursing and Health Sciences, Da-Yeh University, Changhua, Taiwan; 2 Department of Neurology, Yuan Sheng Hospital, Changhua, Taiwan; 3 Department of Neurology, Show Chwan Memorial Hospital, Changhua, Taiwan; 4 Management Office for Health Data, China Medical University Hospital, Taichung, Taiwan; 5 Department of Cardiology, Yuan Rung Hospital, Changhua, Taiwan; 6 Graduate Institute of Clinical Medical Science, China Medical University, Taichung, Taiwan; 7 Department of Health Services Administration, China Medical University College of Public Health, Taichung, Taiwan; 8 Department of Food Nutrition and Health Biotechnology, Asia University, Taichung, Taiwan

**Keywords:** osteoarthritis, ischemic stroke, hypertension, non-steroid anti-inflammatory drugs, aspirin

## Abstract

**Background:**

Osteoarthritis (OA) is more prevalent in women with age. Comorbidities are prevalent in OA patients. In this study, we conducted a follow-up study to evaluate whether women with OA are at an increased risk of ischemic stroke using insurance claims data of Taiwan.

**Methods:**

We identified 13,520 women with OA aged 20–99 newly diagnosed in 2000–2006 and 27,033 women without OA for comparison, frequency matched by age and diagnosis date. Women with baseline history of hypertension and other disorders associated with stroke were excluded for this study. Incident ischemic stroke was assessed by the end of 2013. A nested case-control analysis was used to identify factors associated with the stroke in the OA cohort.

**Results:**

The incidence rate of ischemic stroke in the OA cohort was 1.5-fold greater than that in comparisons (1.93 versus 1.26 per 1,000 person-years), with an adjusted hazard ratio of 1.34 (95% confidence interval [CI], 1.09–1.66). The nested case-control analysis showed that stroke cases were twice as likely to develop hypertension during the follow-up period than controls without stroke. The ischemic stroke risk was significantly associated with hypertension (odds ratio [OR] 1.84; 95% CI, 1.37–2.46) and atrial fibrillation (OR 2.25; 95% CI, 1.24–4.09). Ischemic stroke was not associated with the use of non-steroidal anti-inflammatory drugs or aspirin.

**Conclusion:**

Women with OA are at an elevated risk of ischemic stroke. A close monitoring of hypertension, atrial fibrillation, and other stroke related comorbidities is required for stroke prevention for OA patients.

## INTRODUCTION

Osteoarthritis (OA) is a progressing inflammatory disorder of joint cartilage and underlying bone primarily due to injury. The elderly with this disorder are at an increased risk of disability of multiple joints and impaired quality of life.^1^ There are multiple factors leading to OA, including traumatic injury, aging, obesity, and other biomechanical disorders.^2^^,^^3^ The disease is more prevalent in women than in men, and prevalence increases with age, especially after 50 years of age, maybe due to hormonal change.^4^^–^^7^ OA of the knee and hand are more prevalent than the OA of hip.^6^^,^^8^

Comorbidities are more prevalent in patients with OA than population free of OA.^9^^–^^11^ Among chronic diseases, cardiovascular disease (CVD) is the most prevalent comorbidity in OA patients observed in the clinical practice, but findings are conflicting. A Canadian study found 38.1% of 2,186 OA patients developed CVDs over a median follow-up of 9.2 years, including acute myocardial infarction, coronary revascularization, heart failure, stroke, or transient ischemic attack.^9^ Whereas a case-control study with a large sample size in the Netherlands found a 3-fold higher risk of cerebrovascular accident in OA patients than in controls.^10^ The European multi-center EPOSA Study found that anxiety, depression, stroke, and osteoporosis are common comorbidities in patients with hand osteoarthritis, partially mediate by pain.^12^ Both ischemic stroke and CVD share a similar mechanism of vascular pathogenesis.

Acetaminophen and non-steroidal anti-inflammatory drugs (NSAIDs) are common pain medications used for patients with OA.^11^^,^^13^^,^^14^ The use of NSAID has been associated with elevated CVD risk, such as stroke,^15^^–^^17^ and with the increase of blood pressure.^18^ A systemic study summarized six studies on the risk of stroke associated with using various NSAIDs and reported relative risk estimates in the range 1.04–2.38 among 11 treatments.^16^ In a review article, Snowden and Nelson reported that the systolic blood pressure raised up to 14.3 mm Hg in hypertensive patients taking NSAIDs.^18^ Hypertension is a known risk factor of stroke.

Stroke is one of the leading causes of morbidity and mortality worldwide, and women are at higher risk of stroke than men, particularly for ischemic stroke.^19^^–^^22^ The recurrent rate of stroke is also higher in women than in men, especially after the age of 45–50.^23^ The longer life expectancy and declined neuroprotective effect due to reduced estrogen may increase the stroke risk in women.^24^^,^^25^ Women in the menopausal transition period may experience the reduced level of endogenous oestrogen for 60%.^24^ The Nurses’ Health Study found that using NSAIDs for more than 15 days/month could reduce mean estradiol levels for 10.5% and free estradiol for 10.6% in women.^26^ The risk of stroke may be thus increased in women with OA using NSAIDs. However, the relationship between OA and ischemic stroke in women remains unclear, particular in elderly women. Studies on medication of NSAIDs and the stroke risk in OA patients are also limited. The present study aims to investigate whether women with OA are at an increased risk of subsequent occurrence of ischemic stroke using the National Health Insurance Research Database (NHIRD) of Taiwan. We also investigated the role of using NSAID and aspirin relating to the stroke risk for women with OA.

## METHODS

### Data source

The Taiwan National Health Research Institutes has established several National Health Insurance Research Databases (NHIRDs) from the claims data of the single-payer compulsory insurance program. This study used the Longitudinal Health Insurance Database (LHID) containing claims data of one million insured people randomly selected from 23 million population. The Bioresource Center at NHRI used the skill of linear congruential random number generation to select the 1 million insured population. They randomly selected 50,000 persons as a group and 20 groups were selected. Distributions of sex, age, and income were similar between the 1 million sample and all insured people. (The information is available at http://nhird.nhri.org.tw/date_01.html).

Information on demographic status, inpatient and outpatient care, prescribed medicines, and costs were available in the database from 1996 to 2013. Personal identifications had been re-coded and replaced with surrogate identifications according to the personal information protection act. The research ethics committee of China Medical University and Hospital in Taiwan approved this study (CMUH 104-REC2-115).

### Study subjects

**Figure 1.**
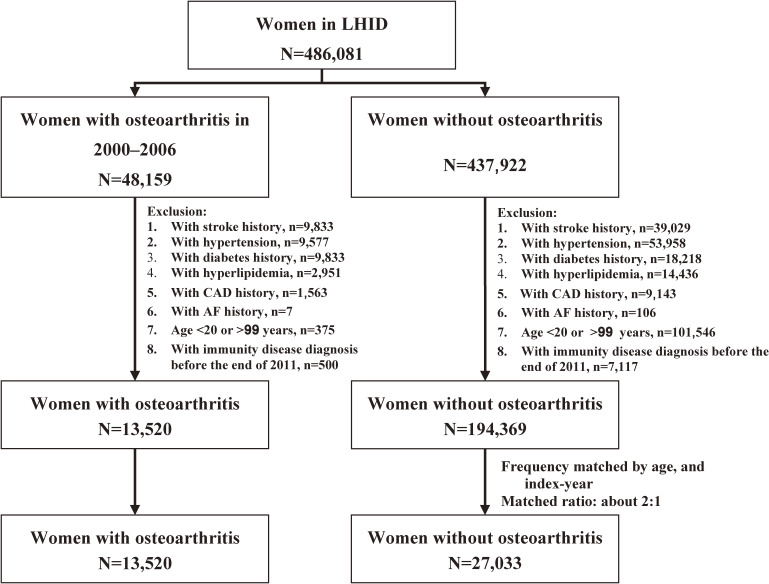
Flow chart of establishing the study cohorts

Women with osteoarthritis (OA) aged 20–99 newly diagnosed in 2000–2006 were identified as the OA cohort using the International Classification of Diseases, Ninth Revision, Clinical Modification (ICD-9-CM) code 715. Patients with baseline history of stroke (ICD-9-CM code 430–438), hypertension (ICD-9-CM code 401–405), diabetes (ICD-9-CM code 250), hyperlipidemia (ICD-9-CM code 272), coronary artery disease (CAD; ICD-9-CM code 410–414), atrial fibrillation (AF; ICD-9-CM code 427.31) and chronic kidney disease (CKD; 580–589) were excluded. Patients who have been diagnosed with immunity disease (ICD-9-CM code 279), systemic lupus erythematous (ICD-9-CM code 710.0), or rheumatoid arthritis (ICD-9-CM code 714.0) at baseline were also excluded. For each OA case, we randomly selected two controls from people without OA, frequency matched by age and diagnosed year, with same exclusions applied (Figure 1).

### Covariates: medication use and morbidity

All study subjects were followed up from the index date until the date ischemic stroke developed (ICD-9-CM code 433, 434, 436, and 437), or withdrew from the insurance program, or by the end of 2013. NSAID and aspirin uses have been associated with the development of adverse cardiovascular events.^5^^,^^22^^,^^27^ Women who had used these medicines for at least 1 year were considered as users. Hypertension, diabetes, hyperlipidemia, AF, CAD, and CKD developed during follow-up period were identified as covariates that might associate with the outcome in the study.

### Statistical analysis

We compared baseline distributions of age group (20–44, 45–64 and ≥65 years), and medicine uses (NSAIDs and aspirin) between the two study cohorts. Chi-square test was used to examine the categorical data and *t*-test was used to examine the difference between means of age. Kaplan-Meier analysis was used to calculate and plot the cumulative incidence of ischemic stroke for both cohorts and log rank test was used to examine the cumulative curves. Cases of incident ischemic stroke developed during the follow-up period were calculated for each cohort and incidence rate was the number of ischemic stroke cases divided by the sum of follow-up person-years. We used Cox proportional hazards regression analysis to calculate the OA cohort to the comparison cohort hazard ratio (HR) of ischemic stroke and 95% confidence interval (CI) by age. Multivariable Cox model was used to calculate adjusted HR (aHR) after controlling for NSAID and aspirin use. A nested case-control analysis was further performed to assess ischemic stroke-associated risk factors for women with OA. The logistical regression analysis was used to assess the odds ratio (OR) of ischemic stroke and 95% CI and related risk factors in the OA cohort. The adjusted odds ratios (aORs) were measured with multivariable logistical regression analysis including age, disorders developed during follow-up period, and the usages of NSAID and aspirin. All statistical analyses used SAS software version 9.4 (SAS Institute, Cary, NC, USA), and the statistical significance level was set *P* < 0.05 at two-tailed test.

## RESULTS

The study population consisted of 13,520 women in the osteoarthritis cohort and 27,033 women in the comparison cohort, with the same mean age of 50.1 years (standard deviation, 12.7 years) (Table 1). The OA cohort had higher medication uses of NSAID (84.8% vs 46.0%) and aspirin (2.41% vs 1.56%) than controls.

**Table 1.** Comparison of baseline distributions of age and uses of NSAIDs and aspirin between osteoarthritis cohort and comparison cohort

	Osteoarthritis cohort		Comparisons		*P*-value
	*N* = 13,520		*N* = 27,033		

	*n*	%	*n*	%	
Age, years					0.97
20–44	4,566	33.7	9,112	33.7	
45–64	7,204	53.3	14,408	53.3	
≥65	1,760	13.0	3,513	13.0	
Mean (SD)	50.1	(12.7)	50.1	(12.7)	0.90
NSAID use	11,463	84.8	12,443	46.0	<0.0001
Aspirin use	326	2.41	422	1.56	<0.0001

NSAID, non-steroidal anti-inflammatory drug; SD, standard deviation.

**Figure 2.**
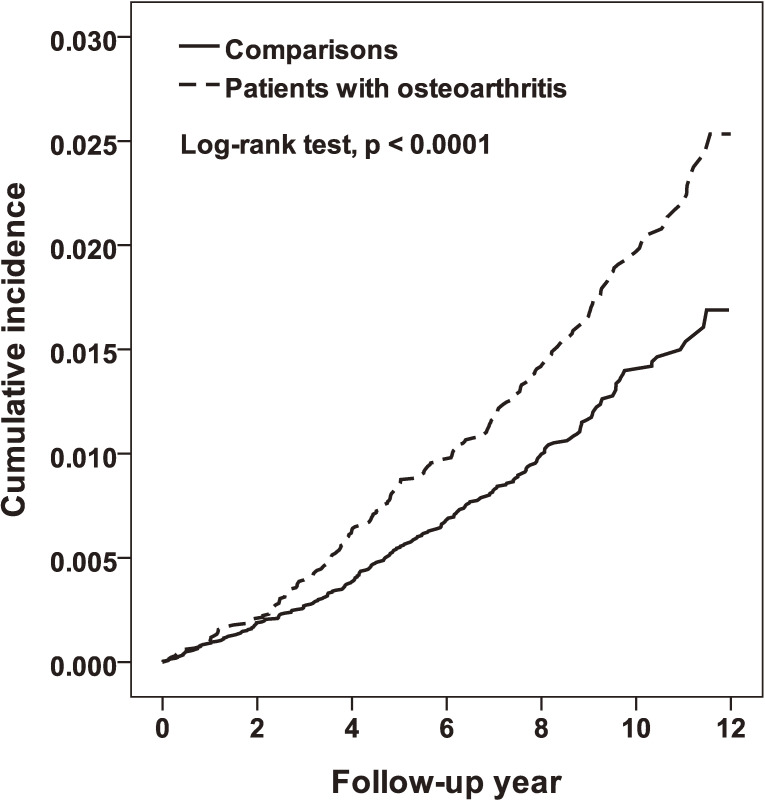
Cumulative incidence of stroke compared between patients with and without osteoarthritis

The cumulative incidence of ischemic stroke in OA patients was significantly higher than that in comparisons (2.53% vs 1.69%) during the study period (Figure 2). The incidence rate of ischemic stroke was near 1.5-fold greater in the OA cohort than in the comparison cohort (1.93 and 1.26 per 1,000 person-years), with an aHR of 1.34 (95% CI, 1.09–1.66) (Table 2). The incidence of ischemic stroke increased with age in both cohorts, but the OA cohort to the comparison cohort HR was the highest for those aged 20–44 years and the lowest for those aged 45–64 years.

**Table 2.** Incidence of ischemic stroke and osteoarthritis cohort to comparison cohort hazard ratio (95% confidence interval) by age

Age	Osteoarthritis cohort			Comparison cohort			Hazard ratio	

	Event	Person-years	Rate, per 1,000 person-years	Event	Person-years	Rate, per 1,000 person-years	Crude	Adjusted
Overall	228	118,425	1.93	237	187,419	1.26	1.46 (1.22–1.75)^***^	1.34 (1.09–1.66)^**^
20–44	18	40,326	0.45	14	71,701	0.20	2.27 (1.13–4.66)^*^	2.15 (1.01–4.60)^*^
45–64	83	63,677	1.30	99	96,719	1.02	1.18 (0.88–1.58)	1.21 (0.87–1.69)
≥65	127	14,422	8.81	124	18,998	6.53	1.24 (0.97–1.59)	1.45 (1.09–1.94)^*^

Rate, per; adjusted model include age, non-steroidal anti-inflammatory drugs, and aspirin.

^*^P < 0.05, ^**^P < 0.01, and ^***^P < 0.001.

Findings in the nested case-control analysis show that the ischemic stroke risk increased with age, with the aOR increased from 2.58 (95% CI, 1.54–4.32) for 45–64 years group to 14.8 (95% CI, 8.81–24.7) for the elderly, compared to those of 20–44 years old (Table 3). Among diseases developed during the follow-up period, ischemic stroke was significantly associated with hypertension (aOR 1.84; 95% CI, 1.37–2.46), and AF (aOR 2.25; 95% CI, 1.24–4.09). Ischemic stroke was not associated with using NSAIDs or aspirin.

**Table 3.** Nested case-control analysis of ischemic stroke in osteoarthritis cohort

Variable	Ischemic stroke				Crude OR (95% CI)	Adjusted OR (95% CI)

	Yes		No			
	*N* = 228		*N* = 13,292			
Age, years	*n*	%	*n*	%		
20–44	18	7.89	4,538	34.1	1.00	1.00
45–64	83	36.4	7,121	53.6	2.94 (1.76–4.90)^*^	2.58 (1.54–4.32)^***^
≥65	127	55.7	1,633	12.3	19.6 (11.9–32.2)^***^	14.8 (8.81–24.7)^***^
Morbidity (yes vs no)						
Hypertension	116	50.9	3,300	24.8	3.14 (2.41–4.08)^***^	1.84 (1.37–2.46)^***^
Diabetes	35	15.4	1,265	9.52	1.72 (1.20–2.48)^**^	1.13 (0.76–1.69)
Hyperlipidemia	55	24.1	2,699	20.3	1.25 (0.92–1.70)	0.97 (0.69–1.37)
AF	14	6.14	148	1.11	5.81 (3.31–10.2)^***^	2.25 (1.24–4.09)^**^
CAD	33	14.5	1,157	8.70	1.78 (1.22–2.58)^**^	0.85 (0.57–1.28)
CKD	15	6.58	599	4.51	1.49 (0.88–2.54)	1.12 (0.65–1.92)
Baseline NSAID use	190	83.3	11,273	84.8	0.90 (0.63–1.27)	1.02 (0.71–1.46)
Baseline aspirin use	4	1.75	322	2.42	0.72 (0.27–1.95)	0.76 (0.28–2.09)

AF, atrial fibrillation; CAD, coronary artery disease; CI, confidence interval; CKD, chronic kidney disease; NSAID, non-steroidal anti-inflammatory drug; OR, odds ratio.

The adjusted OR was measured by including all variables.

## DISCUSSION

Osteoarthritis (OA) is a common bone disease prevalent in the elderly. Patients with OA suffer from joint pain, limiting movement, causing functional decline, and affecting quality of life.^1^^,^^27^^,^^28^ CVD is a common comorbidity in patients with OA, whereas the relationship between OA and stroke risk has not yet clearly been established. Our population-based follow-up study revealed women with OA had an aHR of 1.34 for the ischemic stroke compared to women without OA. The age-specific incidence of ischemic stroke increased with age in both cohorts. The age-specific difference in the stroke incidence between the OA cohort and comparison cohort also increased with age, from 0.25 per 1,000 person-years in younger women to 2.28 per 1,000 person-years in the elderly. The stroke risk associated with OA is likely greater for the elderly. However, the OA cohort to comparison cohort HR was the greatest in 20–44 years group. The HR declined to insignificant level in 45–64 years group; this was because the incidence of stroke in comparisons had a 5.1-fold increase, which was much greater than the OA group, which had a 2.89-fold increase.

Pain is the predominant symptom in OA patients, affecting physiologic function and limiting physical movements.^28^ The therapy of OA may involve non-pharmacologic and pharmacologic therapies. Among pharmacological modalities, both NSAIDs and aspirin are the most frequently used agents for rapid pain relief.^11^^–^^14^^,^^27^^–^^31^ NSAIDs may account for 73% prescriptions in OA patients.^21^ Our study shows that nearly 85% of OA patients used NSAIDs, whereas only 2.41% patients used aspirin.

Studies have associated NSAIDs with increased adverse effects on ischemic cardiomyopathy.^32^^,^^33^ Our nested case-control analysis failed to show the risk of ischemic stroke in relating to the use of NSAIDs or aspirin. This finding is consistent with an Italian nested case-control study, which found no increased cerebrovascular event for current NSAIDs users.^34^ A population-based case-control study in Spain also failed to find ischemic stroke is associated with the use of ibuprofen, naproxen, and aspirin.^35^ On the other hand, studies have found the increased ischemic stroke risk in NSAIDs users is associated with other disorders in the users.^16^^,^^17^ After reviewing studies, Park and Bavry concluded that hypertension and thrombosis are the contributors of stroke in NSAIDs users.^16^ A case-crossover study also using Taiwan’s insurance claims data found the increased ischemic stroke risk is related to hypertension in NSAIDs users.^17^ Our nested case-control analysis revealed the stroke cases developed more incident disorders than controls during the follow-up period. The ischemic stroke risk is significantly associated with age, hypertension, and AF. Half of stroke cases were hypertensive, which was twice the incidence of controls. Cases had a 4.5-fold higher proportion of the elderly women than controls had (55.7% vs 12.3%). More than 80% of OA women used NSAID and were similar in cases and controls, whereas aspirin was rarely used. The data reflect that the ischemic stroke risk was mainly associated with disorders relating to stroke, developed when women with OA were aging during the follow-up period. Hypertension and AF are two powerful risk factors of ischemic stroke among those known risk factors.^36^^–^^39^

Mechanisms that explain the ischemic stroke risk for women with OA may associate with the progress of inflammation and the change of physical activity. The chronic inflammation of OA is a mediator of oxidative stress because of the activation of Renin Angiotensin System (RAS), leading to endothelial dysfunction and atherosclerosis.^40^ Patients with OA tend to have reduced physical activity because of the pain. Immobility resulting from OA can increase heart disease or stroke risk to shorten their life expectancy.^41^^–^^43^ OA patients with limited physical activity are more likely overweight or obese, at an increased risk of developing hypertension and other cardiovascular disorders associating with ischemic stroke.^9^^,^^41^^–^^44^ In a population-based follow-up study with 1,163 OA patients, Nuesch et al found that patients with walking disability are at a higher mortality risk associated with mainly cardiac disorders.^41^

We have conducted a further data analysis to assess the risk of hemorrhagic stroke. The results showed that the incidence of hemorrhagic stroke was slightly lower in the OA cohort than in the comparisons (0.53 vs 0.71 per 1,000 person-years; or 85 cases/135,707 person-years vs 154 cases/218,324 person-years). This finding indicates that women with OA are at slightly lower risk of hemorrhagic stroke. Further nested case-control analysis revealed the hemorrhagic stroke was strongly associated with hypertension developed during the follow-up (aOR 4.82; 95% CI, 2.82–8.23), but not AF (aOR 0.48; 95% CI, 0.12–2.01).

The present study is the first longitudinal study on the ischemic stroke risk in women with OA. This study is strengthened by using claims data with a large representative population.^45^ However, there are limitations in our study. First, the information on the severity of OA related pain was not available from the claims data for evaluating the ischemic stroke risk by the severity of OA pain. Patients with mild pain might not seek medical care and were not included in the OA cohort. Second, information on white blood cell count, high sensitivity C-reactive protein, and data of other inflammatory biomarkers and biochemistry data were also not available in the claimed database to assess the association with stroke. Third, NSAIDs and aspirin are over-the-counter and available without prescription in Taiwan. However, these medications are generally prescribed by physicians for patients without additional cost. This is particularly true for patients with OA, of whom nearly 85% were prescribed. It is likely that the over-the-counter usage might be low. Fourth, patients with AF tend to be pre-treated with Novel Oral Anticoagulants (NOAC) or anticoagulant drug in clinic. However, in the present study, we established study cohorts by excluding study subjects with baseline history of stroke, hypertension, diabetes, hyperlipidemia, coronary arterial disease, and AF. Therefore, our study population is not likely pretreated by NOACs. Fifth, lifestyle factors, such as smoking, drinking, and exercise habit and body weight are potential risk factors of stroke.^46^^–^^51^ The information was also not available in the claims data, and thus could not be further adjusted in this study. However, the impact of these variables on stroke in this study is likely minor because less than 5% of women in Taiwan smoke and/or drink.^52^

### Conclusions

In this longitudinal study, we found that women with OA are at an elevated risk of ischemic stroke, with a greater impact observed in younger patients and the elderly. The nested case-control analysis within the OA cohort revealed that a much higher portion of women had developed cardiovascular risk conditions in ischemic stroke cases than in controls. Hypertension and AF developed during the follow-up period were important risk factors associated with stroke. Our findings imply that OA patients should be closely monitored for the development of cardiovascular disorders, particularly for hypertension and atrial fibrillation, to prevent stroke.
